# Underlying Security Transmission Design for Orthogonal Time Frequency Space (OTFS) Modulation

**DOI:** 10.3390/s22207919

**Published:** 2022-10-18

**Authors:** Wei Liang, Xuan Liu, Jia Shi, Lixin Li, Junfan Hu

**Affiliations:** 1 School of Electronics and Information, Northwestern Polytechnical University, Xi’an 710072, China; 2 State Key Laboratory of Integrated Services Networks, Xidian University, Xi’an 710071, China

**Keywords:** secure transmission, OTFS, security analysis

## Abstract

With the aim of ensuring secure transmission in high-mobility wireless scenarios, this paper proposes a 2D permutation-aided Orthogonal Time Frequency Space (OTFS) secure transmission scheme, which uses the Gosudarstvennyi Standard (GOST) algorithm to perform disturbance control on the OTFS modulation domain. Furthermore, we develop an improved SeLective Mapping (SLM) algorithm, which can significantly improve the Peak-to-Average Power Ratio (PAPR) problem with very low complexity. In addition, we carry out the security analysis, investigating the proposed scheme’s resistance performance to a range of effective attacks. Finally, our numerical results show that our proposed transmission scheme can guarantee the underlying security property of OTFS.

## 1. Introduction

As a mobile network, 6G will be a fully connected world integrating terrestrial wireless networks [1]. It is no longer a simple breakthrough in network capacity and transmission rate but the realization of the interconnection of everything [2]. As a result, many new high-mobility wireless services have emerged, such as the Low Earth Orbit (LEO) communication satellite, unmanned aerial vehicle (UAV) communications, high-speed rail communications, and Internet of Vehicles communications [3]. However, these new wireless services all have the characteristics of high mobility, which will inevitably bring a severe Doppler effect problem. Due to the additional frequency components introduced by Doppler spread, it will result in severe inter-symbol crosstalk and a huge degradation of link reliability.

Orthogonal Frequency Division Multiplexing (OFDM) technology has been widely used in 5G mobile networks due to its high spectrum utilization [4]. OFDM technology combines frequency diversity and time diversity organically, which not only can greatly improve channel capacity but also can effectively resist multipath fading and interference [5]. However, OFDM transmissions have poor reliability performance in high-mobility scenarios, in which the high Doppler frequency shift can easily destroy the orthogonality between OFDM subcarriers. Although the OFDM in the 5G system adopts a larger and more flexible subcarrier spacing design, the increase in the subcarrier spacing will shorten the Cyclic Prefix (CP) and reduce the anti-multipath capability, as well as degrade the spectrum efficiency [6]. Furthermore, the channel response in a high-mobility environment presents fast time-varying and non-stationary characteristics, which greatly increase the overhead for channel estimation under the OFDM scheme. Recently, R. Hadani et al. proposed a new modulation method, namely Orthogonal Time Frequency Space (OTFS) modulation, for combating Doppler spread in high-mobility scenarios. In particular, the OTFS modulation scheme places the data in the Delay Doppler (DD) domain, which is relatively insensitive to time changes, and spreads each symbol in the entire Time Frequency (TF) domain by the Inverse Symplectic Finite Fourier Transform (ISFFT) so that each OTFS symbol experiences almost the same channel gain.

The OTFS technique has drawn a lot of research attention, such as [7,8,9,10,11,12,13]. In particular, refs. [7,8,9] were devoted to researching the Peak-to-Average Power Ratio (PAPR) of the OTFS system, wherein [7] proposed a MuLaw companding technique to reduce the PAPR of the OTFS, and [9] proposed an effective PAPR reduction method based on an iterative limiting and filtering framework. The channel estimation methods designed for OTFS transmission were studied in [10,11,12]. In a little more detail, ref. [10] proposed an algorithm based on Orthogonal Matching Pursuit (OMP) and Modified Subspace Pursuit (MSP) for the DD channel estimation in OTFS multiple access (OTFS-MA) systems on the uplink. In Multiple-Input Multiple-Output (MIMO) scenarios based on the OTFS, ref. [11] proposed an iterative signal detection algorithm based on message passing and a channel estimation scheme in the DD domain. An embedded pilot-assisted channel estimation scheme was proposed in [12].

Under the OTFS scheme, especially for future high-mobility scenarios, secure transmission is particularly important and still faces huge challenges. As is known, the future 6G mobile network contains a range of high-mobility scenarios, such that UAV communications are deployed to supplement the terrestrial networks, building an integrated air–space–earth–sea network to cover a much wider area. In this case, the probability of eavesdropping on the communication links significantly increases, and thus there is a greater risk of information exposure. Nevertheless, as far as we know, very limited studies [13,14] have been devoted to the security issue of OTFS transmissions.

To ensure the security of wireless communications, traditional security mechanisms based on cryptography are mainly deployed on multilayer protocols above the physical layer, and the main idea is to improve the security of the wireless system by using a variety of upper layer authentication mechanisms. For example, at the Medium Access Control (MAC) layer, the MAC address is authenticated so that access is only granted to legitimate users. At the network layer, two authentication protocols for the wireless Local Area Network (LAN) security access WPA and WPA2 were adopted [15,16]. Although upper layer encryption can enhance the security of the wireless system, the corresponding techniques are completely dependent on the confidentiality of the key and the computational complexity. However, with the continuous development of quantum computing technology and breakthroughs in computing power, traditional cryptographic schemes may be easily broken, and they face great challenges. Moreover, one of the prerequisites for upper layer encryption is that the physical layer can actively provide smooth and error-free transmission, which is difficult to achieve in practical scenarios [17]. In contrast, physical layer security techniques can compensate well for the shortcomings of traditional encryption schemes and have gradually gained widespread attention in recent years. Compared with traditional encryption methods, without considering the computational power of attackers, the physical layer security technology can make full use of the instantaneity and randomness of wireless channels to achieve secure transmission without sharing the key between the legitimate transmitter and receiver. The principle of physical layer key generation technology is to use the reciprocity, time variability, and spatial uniqueness of wireless channels to generate the key. Depending on the randomness of a wireless channel, the key generation schemes are mainly divided into the following four types: Channel State Information (CSI)-based key generation technology [18], signal strength-based key generation technology [19], phase-based key generation technology [20], and eavesdropping encoding key generation technology [21]. Further, ref. [22] proposed the key generation scheme under active attack, which used random detection signals to combine the user-generated randomness and channel randomness. Ref. [23] proposed an adaptive channel detection scheme based on a proportional-integral-derivative controller, which can achieve the desired Key Generation Rate (KGR) by adjusting the detection rate.

As far as we know, the current research on OTFS secure transmission is still in its infancy. In particular, ref. [13] studied the secrecy performance of an OTFS-based uplink LEO Sat-Com system, where a cooperative UAV was employed to send jamming signals against the reconnaissance satellite. However, the study only targeted the uplink LEO Sat-Com scenario and is not universal. Ref. [14] studied the security performance of the unicast–multicast streaming system by deriving and analyzing the maximum secrecy rate and positive secure capacity probability (PSCP) of unicast transmission. Ref. [24] proposed a novel physical layer security transmission scheme by encrypting the Discrete Fourier Transform (DFT) matrix to guarantee the underlying security property of the OFDM. It is very unfortunate that there is currently no research on physical layer security via control of the modulation matrix in the OTFS. Against the background above, we are motivated to study secure transmission by controlling the modulation matrix for the OTFS transmission in high-mobility communication scenarios. Our key contributions are summarized as follows:

- We propose a 2D permutation OTFS secure transmission scheme that uses the Gosudarstvennyi Standard (GOST) algorithm to perform disturbance control on the OTFS modulation domain so as to realize the underlying security of the OTFS transmission. By utilizing the instantaneities and uniquenesses of wireless channels, the GOST algorithm can generate a control sequence to perturb the ISFFT matrix under OTFS modulation.
- We propose a so-called Selective Mapping (SLM) algorithm, which can significantly improve the PAPR problem of our secure OTFS transmission. With very low complexity, the SLM algorithm aims to select the best transition matrix to multiply with the TF domain signal for the sake of ensuring the low PAPR characteristic.
- We conduct a security analysis for our OTFS secure transmission. We prove our scheme has a promising resistance performance to a range of effective attacks, including brutal force, chosen-plaintext, statistical attacks, etc.
- We carry out a comprehensive simulation evaluation. Our experimental results show that our proposed transmission scheme can guarantee the underlying security property of the OTFS. Furthermore, compared to the OFDM scheme, our proposed scheme can reduce the PAPR by 20% and improve the BER by at least 4 dB under the same security requirements.

The rest of this paper will be organized as follows: In Section 2, we briefly introduce the OTFS system model. In Section 3, we propose the design for a 2D permutation-aided OTFS secure transmission scheme. In Section 4, we introduce the principle of the key generation algorithm and analyze its performance. In Section 5, we conduct a security performance analysis of the proposed transport mechanism. In Section 6, the performance of the design of our 2D permutation-aided OTFS secure transmission is evaluated. Finally, Section 7 concludes the paper.

## 2. System Model

We assume a classic three-node secure communication model, in which the transmitter (Alice) transmits private information to the legitimate receiver (Bob) with the risk of being passively eavesdropped by an illegal eavesdropper (Eve). We assume that Alice is in a state of high mobility and that Bob and Eve are static. All nodes are equipped with a single antenna. Let us assume that each communication link has *L* propagation paths. The channel response of the high-mobility channels can be given by

(1) $$h\left(\tau ,\nu \right)=\sum_{p=1}^{L}h_{p}\delta \left(\tau -\tau_{p}\right)\delta \left(\nu -\nu_{p}\right),$$

where $\tau_{p}=\frac{l_{p}}{M△f}$ and $\nu_{p}=\frac{k_{p}}{NT}$ denote the delay and the Doppler shift, respectively, and $h_{p}$ is the Rayleigh fading.

To overcome the Doppler effect in a high-mobility scenario, Alice transmits signals to Bob based on the OTFS scheme. Specifically, the signal in the DD domain is denoted by $x\left[k,l\right]$, where $k\in \left\{0,\cdots ,N-1\right\}$. *N* represents the number of Doppler bins, $l\in \left\{0,\cdots ,M-1\right\}$, and *M* represents the number of Delay bins. Firstly, the symbols $x\left[k,l\right]$ are preprocessed by using the Inverse Symplectic Finite Fourier Transform (ISFFT), thereby deriving the TF domain signal as

(2) $$X\left[n,m\right]=\frac{1}{\sqrt{NM}}\sum_{k=0}^{N-1}\sum_{l=0}^{M-1}x\left[k,l\right]e^{j2\pi \left(\frac{nk}{N}-\frac{ml}{M}\right)},$$

where $n\in \left\{0,\cdots ,N-1\right\}$ and $m\in \left\{0,\cdots ,M-1\right\}$. Then, by applying the Heisenberg transform, the signal $X\left[n,m\right]$ is converted into the time domain signal. Assume that the sending and receiving pulses satisfy the bi-orthogonal property. After applying the Wigner transform, the signal in the TF domain received by Bob can be expressed as

(3) $$Y\left[n,m\right]=H\left[n,m\right]X\left[n,m\right]+W\left[n,m\right],$$

where W[n,m] is the complex Gaussian distributed noise in the TF domain, and $H\left[n,m\right]=\;\int \int \;h\left(\tau ,\nu \right)e^{j2\pi \nu nT}e^{-j2\pi (\nu +m\Delta f)\tau }d\tau d\nu$. By using the Symplectic Finite Fourier Transform (SFFT), the signal $Y\left[n,m\right]$ in the TF domain is transformed into the signal $y\left[k,l\right]$ in the DD domain:

(4) $$y\left[k,l\right]=\frac{1}{\sqrt{NM}}\sum_{n=0}^{N-1}\sum_{m=0}^{M-1}Y\left[n,m\right]e^{-j2\pi \left(\frac{nk}{N}-\frac{ml}{M}\right)}.$$

## 3. 2D Permutation-Aided OTFS Secure Transmission

In this section, we propose a so-called 2D permutation-aided OTFS secure transmission scheme, which aims to generate control sequences by utilizing the instantaneities and uniquenesses of wireless channels. Then, the control sequence is used to perturb the OTFS modulation domain, which ensures the underlying security of the OTFS transmission. Furthermore, we develop the SLM algorithm to guarantee the low PAPR feature of the OTFS secure transmission.

### 3.1. Design of 2D Permutation-Aided OTFS Secure Transmission

**Figure 1:**
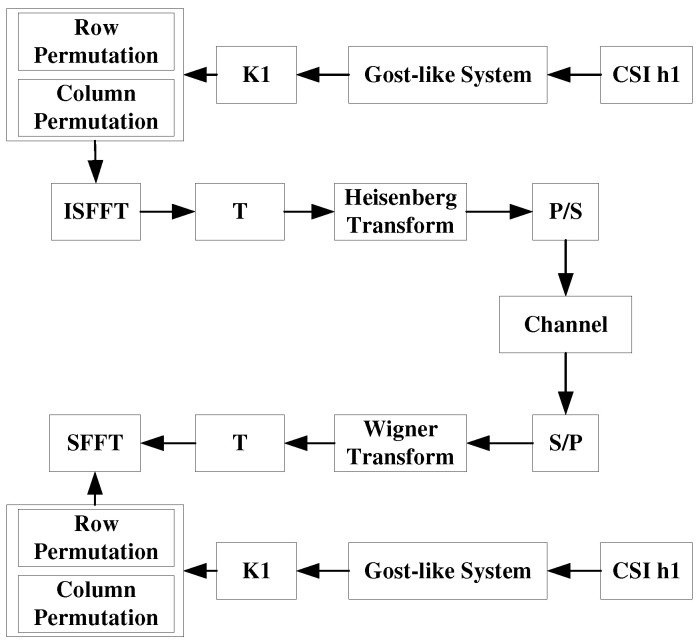
Flowchart for 2D permutation-aided OTFS secure transmission.

As shown in Figure 1, the proposed OTFS secure transmission is mainly divided into four stages: (1) security sequence generation, (2) interleaving replacement, (3) signal modulation, and (4) signal demodulation.

- *security sequence generation*

Alice and Bob detect the channel information through channel measurement and use the channel information as the input of the GOST method (detailed in Section 4)-based secret key generation algorithm to generate the index sequence (secret key) that controls the permutation, denoted by $D_{x}$, $D_{y}$, $D_{z}$, $D_{w}$.

- *interleaving replacement*

According to the index sequence, row permutation and column permutation are performed on the IFFT matrix and the FFT matrix, respectively, as follows:

(5) $$\begin{array}{cc} F_{M*M} & =\frac{1}{M}e^{-j2\pi \left(\alpha -1\right)\left(\beta -1\right)/M}\\ \; & =\left[w_{0}^{T},w_{1}^{T},\cdots ,w_{M-1}^{T}\right], \end{array}$$

where α and β are the row index and column index of the standard FFT matrix, and $w_{0}^{T},w_{1}^{T},\cdots ,w_{M-1}^{T}$ are the row vectors. The index vectors $D_{x}$ and $D_{y}$ are used to replace the rows and columns of the standard FFT matrix. The new FFT matrix can be obtained by the following two steps. During the first step, the rows of the FFT matrix are permutated by the index vectors $D_{x}$. The new FFT matrix can be expressed as

(6) $$\begin{array}{cc} F_{M*M}^{{}^{\prime }} & =\left[w_{D_{x}\left(0\right)}^{T},w_{D_{x}\left(1\right)}^{T},\cdots ,w_{D_{x}\left(M-1\right)}^{T}\right]\\ \; & =\left[r_{0},r_{1},\cdots ,r_{M-1}\right]. \end{array}$$

During the second step, the columns of the FFT matrix are permutated by the index vectors $D_{y}$. Then, the modified FFT matrix can be expressed as

(7) $$\begin{array}{cc} F_{M*M}^{{}^{\prime \prime }} & =\left[r_{D_{y}\left(0\right)}^{T},r_{D_{y}\left(1\right)}^{T},\cdots ,r_{D_{y}\left(M-1\right)}^{T}\right]\\ \; & =\left[c_{0}^{T},c_{1}^{T},\cdots ,c_{M-1}^{T}\right]. \end{array}$$

**Figure 2:**
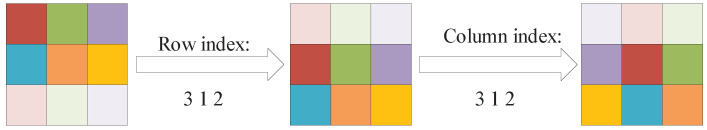
Flowchart of matrix permutation.

To describe row permutation and column permutation more clearly, an example for a 3×3 matrix is given in Figure 2. Assuming that $D_{x}=\left(3,1,2\right)$ and $D_{y}=\left(3,1,2\right)$, the entire permutation process is described as follows. Firstly, perform row permutation by using the row index sequence $D_{x}$. Secondly, perform column permutation by using the column index sequence $D_{y}$.

The modified IFFT matrix is obtained by repeating the above steps using the index vector $D_{z}$, $D_{w}$.

- *OTFS modulation*

The signal $x\left[k,l\right]$ in the DD domain is converted into the signal $X\left[n,m\right]$ in the TF domain, as follows:

(8) $$X\left[n,m\right]=P_{ISFFT}\left(\frac{1}{\sqrt{NM}}\sum_{k=0}^{N-1}\sum_{l=0}^{M-1}x\left[k,l\right]e^{j2\pi \left(\frac{nk}{N}-\frac{ml}{M}\right)}\right),$$

where $n\in \left\{0,\ldots ,N-1\right\}$, $m\in \left\{0,\ldots ,M-1\right\}$, $P_{ISFFT}\left(\cdot \right)$ denotes the ISFFT transform after the FFT matrix and IFFT matrix permutate. Then, let us multiply the TF domain signal $X\left[n,m\right]$ by a multiple *U* group of conversion matrices $T\in \left\{T_{1},T_{2}\cdots ,T_{U}\right\}$, as follows:

(9) $$X_{i}\left[n,m\right]=T_{i}X\left[n,m\right],$$

where i=1,2,⋯U, $T_{i}$ is the *i*-th conversion matrix. Note that $T_{i}=\left[t_{i},t_{i}^{\langle 1\rangle },\cdots ,t_{i}^{\langle N-1\rangle }\right]$. $t_{i}^{\langle 1\rangle }$ is obtained by cyclically shifting $t_{i}$, in which $t_{i}=[p_{1},0,\cdots ,0,p_{2},0,\cdots ,0,p_{3},0,\cdots ,$$0,p_{4},0,$$\left.\cdots ,0\right]$ [25], and then generating a multiple *U* group of time-domain candidate signals through the Heisenberg transform. After this, it selects the group with the smallest PAPR for transmission, as follows:

(10) $$s_{i}\left(t\right)=\sum_{n=0}^{N-1}\sum_{m=0}^{M-1}X_{i}\left[n,m\right]g_{tx}\left(t-nT\right)e^{jz\pi △f\left(t-nT\right)},$$

(11) $$s\left(t\right)=min\left(s_{1}\left(t\right),s_{2}\left(t\right),\cdots ,s_{U}\left(t\right)\right),$$

where $g_{tx}\left(t\right)$ is the transmission pulse shape. This can be seen as a two-dimensional extension of the OFDM modulation transform. When the input signal $s\left(t\right)$ passes through the wireless channel, it will be affected by the channel delay and Doppler shift, as follows:

(12) $$r\left(t\right)=\int \int h\left(\tau ,\nu \right)s\left(t-\tau \right)e^{j2\pi \nu \left(t-\tau \right)}d\tau d\nu +w\left(t\right),$$

where $w\left(t\right)$ denotes the white Gaussian noise, $h\left(\tau ,\nu \right)=\sum_{p=1}^{L}h_{p}\delta \left(\tau -\tau_{p}\right)\delta \left(\nu -\nu_{p}\right)$.

- *OTFS demodulation*

By using the Wigner transform, which obtains the mutual ambiguity function and then performs sampling, the received signal $r\left(t\right)$ in the time domain is converted into the TF domain signal, denoted by $Y{\left[n,m\right]}^{{}^{\prime }}$, as follows:

(13) $$Y{\left[n,m\right]}^{{}^{\prime }}=\int g_{rx}\left(t-\tau \right)e^{-j2\pi \nu \left(t-\tau \right)}r\left(t\right)dt{|}_{\tau =nT,\nu =m△f},$$

where $g_{rx}\left(t\right)$ is the reception pulse shape. Assuming that the transmit pulse $g_{tx}\left(t\right)$ and the receive pulse $g_{rx}\left(t\right)$ are ideal, we have $\int e^{-j2\pi m△f\left(t-nT\right)}g_{rx}\left(t-nT\right)g_{tx}\left(t\right)dt=\delta \left(m\right)\delta \left(n\right)$. Since the TF domain signal is multiplied by the conversion matrix before the Heisenberg transform, the corresponding inverse transformation must be performed after the Wigner transform to obtain the correct DD domain signal $Y\left[n,m\right]$. Then, by using the SFFT, the signal $Y\left[n,m\right]$ in the TF domain is transformed into the signal $y\left[k,l\right]$ in the DD domain:

(14) $$y\left[k,l\right]=P_{SFFT}\left(\frac{1}{\sqrt{NM}}\sum_{n=0}^{N-1}\sum_{m=0}^{M-1}Y\left[n,m\right]e^{-j2\pi \left(\frac{nk}{N}-\frac{ml}{M}\right)}\right),$$

where $k\in \left\{0,\cdots ,N-1\right\}$ and $l\in \left\{0,\cdots ,M-1\right\}$. Further, $P_{SFFT}\left(\cdot \right)$ denotes the SFFT transform after the FFT matrix and IFFT matrix de-permutate.

### 3.2. SLM Algorithm for PAPR Reduction

**Figure 3:**
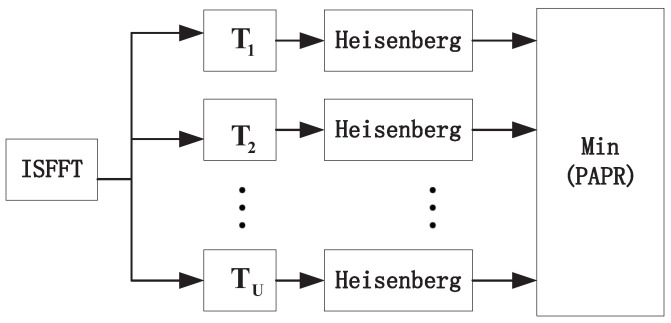
Flowchart of SLM.

To realize secure OTFS transmission, we need to carefully deal with the PAPR problem to avoid eavesdropping. For this reason, we apply the SLM algorithm to our OTFS transmission. In particular, it generates multiple sets of candidate sequences by multiplying the QAM symbols with the *U* groups of the phase rotation vectors and selects the time-domain signal with the lowest peak-to-average power ratio as the transmission sequence, which requires *U* ISFFT transforms and Heisenberg transforms. To reduce the computational complexity, we generate candidate sequences by multiplying the time-frequency domain signal sequence by the transition matrices. The principle of the SLM algorithm developed is described in Figure 3:

Assuming that the OTFS system contains *M* symbols and *N* subcarriers, *x* represents the input signal in the DD domain, *X* represents the signal in the TF domain, $Q_{1}$ is an N×N IFFT matrix, and $Q_{2}$ is an M×M FFT matrix. Then, we have $X=ISFFT\left(x\right)=Q_{1}xQ_{2}$. There are *U* different phase rotation vectors, and the representation is as follows:

(15) $$\gamma_{i}=\left[\begin{array}{cccc} P_{0}^{i} & P_{0}^{i} & \cdots & P_{0}^{i}\\ P_{1}^{i} & P_{1}^{i} & \cdots & P_{1}^{i}\\ \vdots & \vdots & \ddots & \vdots \\ P_{N-1}^{i} & P_{N-1}^{i} & \cdots & P_{N-1}^{i} \end{array}\right],$$

where i=1,2,⋯,U, $P_{b}^{i}=e^{j\varphi_{b}^{i}}$, $\varphi_{b}^{i}$ follows a uniform distribution between $\left[0,2\pi \right]$. Suppose that the ISFFT transform is used to change *X* and $X_{i}$ into S=x and $S_{i}=R_{i}x$, respectively, where $R_{i}$ is the phase transformation matrix corresponding to $\gamma_{i}$. Therefore, $X=ISFFT\left(x\right)=Q_{1}xQ_{2}$, $X_{i}=ISFFT\left(x\right)=Q_{1}R_{i}xQ_{2}$. Given that $T_{i}=Q_{1}R_{i}Q_{1}^{-1}$, we have

(16) $$T_{i}=Q_{1}R_{i}Q_{1}^{-1}=\left[p_{i},p_{i}^{\langle 1\rangle },p_{i}^{\langle 2\rangle },\cdots p_{i}^{\langle N-1\rangle }\right],$$

where $p_{i}^{\langle k\rangle }$ is obtained by circularly shifting $p_{i}$ down by *k* elements. As shown in [25], by choosing the appropriate γ, the complexity of SLM can be significantly reduced.

## 4. Secret Key Generation Algorithm

In our proposed secure OTFS transmission scheme, the key generation algorithm plays the core role. In this section, we propose the key generation algorithm, named GOST, as well as provide an analysis of its characteristics.

### 4.1. Gost Method-Based Secret Key Generation

**Figure 4:**
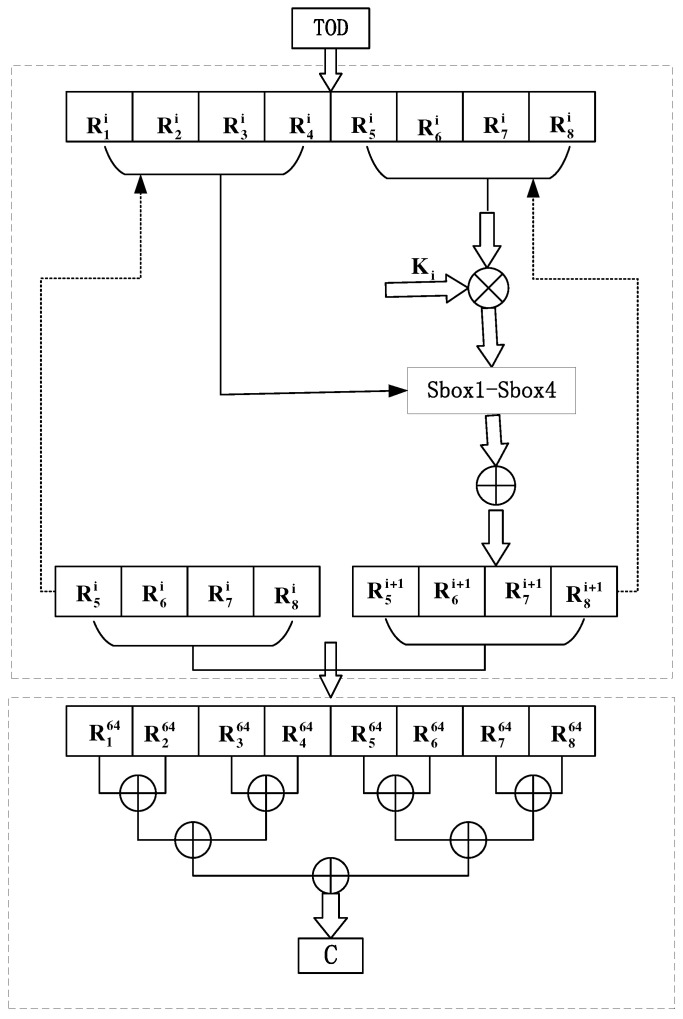
Flowchart for the GOST method-based secret key generation.

As shown in Figure 4, the GOST-based key generation algorithm contains two parts: round function and compression–expansion.

The detailed Gost algorithm is detailed in Algorithm 1. Since our algorithm uses 64-bit grouping on the input data, we must first change the input data to a multiple of 64. That is, if the length of the data to be encrypted is less than 64 bits, it must be padded to 64 bits; if the length of the data to be encrypted exceeds 64 bits, it must first be padded to a multiple of 64. Then, the data that need to be encrypted are divided into two parts, each containing 32 bits. Because the round function is iterated for 64 rounds, we use a 2048-bit key and perform the addition of the (mod$2^{32}$) operation with the right part of the data to obtain the new right part, denoted by itmp. Then, the itmp passes through the S box. That is, the 32-bit itmp is equally divided into eight blocks; each block has four bits, and the value of each block does not exceed Oxf. The S box of the secret key generation algorithm has a total of eight groups that correspond to these eight blocks one-to-one. For example, if the value of the third block is eight, then replace the original value of the third block with the value at the eighth column of the third group of the S box. Repeat the above steps eight times to obtain a new itmp. Subsequently, the new itmp and the left part perform the XOR operation to obtain the right part of the next round. Additionally, the left part of the next round is obtained from the right part of the previous round, as follows:

(17) $$R_{j+4}^{i+1}=Sboxj\left(R_{j+4}^{i+1}⊗K_{i}\right)⊕R_{j}^{i},$$

(18) $$R_{j}^{i+1}=R_{j+4}^{i},$$

where i=1,2,⋯,64 represents the i-th iteration, j=1,2,3,4 represents the j-th S box, ‘⊗’ and ‘⊕’, respectively, represent the addition of (mod$2^{32}$) and the binary operation of XOR. At this point, one round of non-linear transformation is complete. Repeat the above steps. The output ciphertext composed of eight parts can be obtained after 64 iterations. Then, by applying the rotate left operator, the final ciphertext can be obtained, as shown in Equation (19).

(19) $$\begin{array}{cc} C & =\left(\left(R_{1}^{64}⊕R_{2}^{64}\right)⊕\left(R_{3}^{64}⊕R_{4}^{64}\right)\right)\\ \; & ⊕\left(\left(R_{5}^{64}⊕R_{6}^{64}\right)⊕\left(R_{7}^{64}⊕R_{8}^{64}\right)\right) \end{array}.$$

**Algorithm 1:** GOST Method-Based Secret Key Generation.   **Input:** Channel state information *H*.   **Initialize:**
R=zeros(8,8);
tod=d2b(H,64);   for n=1→size(R,1);   todex(n,:)=tod((n−1)∗8+1:(n−1)∗8+8);   end for;   for i=0→63;   First, $R_{right}$ performs mod$2^{n}$ with key;   second, $R_{right}$ passes through the S box;   Third, $R_{right}$ and $R_{left}$ perform XOR operation;   Fourth, update $R_{right}$ and $R_{left}$ following funcation (17) and funcation (18),   respectively;   end for;   Apply the rotate left operator following funcation (19).

**Figure 5:**
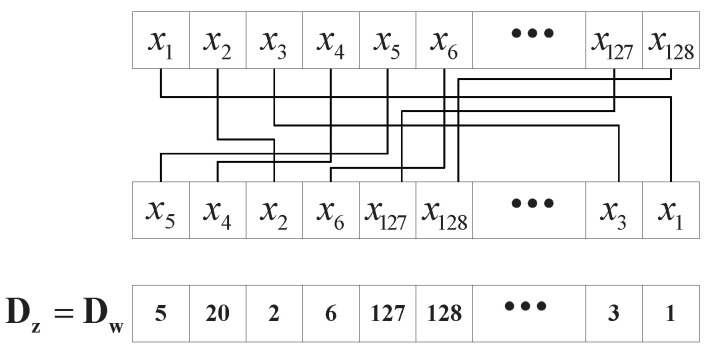
Flowchart of obtaining permutation index vector.

In this article, since we use the detected channel state information as the input of this secret key generation algorithm, we can obtain multiple corresponding output ciphertexts. Using N=128 as an example, in order to control the permutation of the IFFT matrix of the ISFFT transformation, we obtain 128 ciphertext outputs. Sort the output ciphertext in ascending order and then obtain the index sequence ($D_{z}$, $D_{w}$) that controls the row permutation and column permutation, respectively, as shown in Figure 5.

### 4.2. Characteristic Analysis for Secret Key

The performance of the key can directly determine the security performance of the OTFS transmission. In this section, the main performance tests are for randomness and complexity. Let us first clarify the testing conditions as follows. The length of the sequence is L = 10,000. In each test, we select five sets of data streams for the GOST algorithm, the DES algorithm, and the chaotic mapping algorithm, ref. [26] respectively.

#### 4.2.1. Lempel–Ziv Complexity

**Figure 6:**
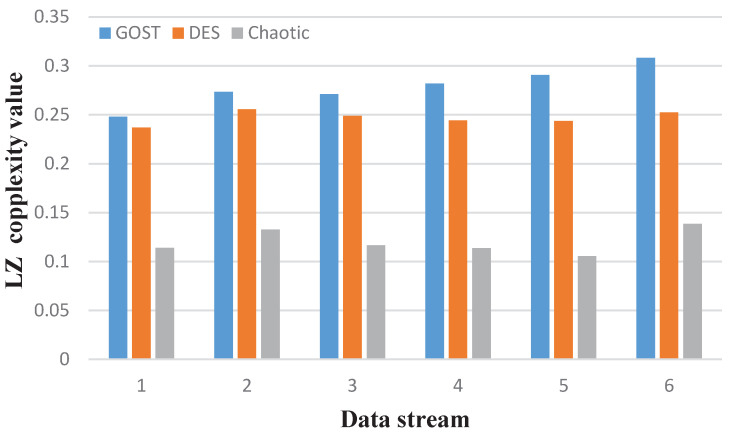
LZ complexity simulation results.

The Lempel–Ziv (LZ) complexity is a simple algorithm for calculating sequence complexity by calculating the rate at which new patterns appear in a sequence. It was first proposed by Lempel and Ziv. Since this method is applicable to symbol sequences, the data sequence must be quantized in advance. That is, the average value of all data must be calculated. Data larger than the average value are set to one, and data smaller than the average value are set to zero. The brief idea of LZ complexity is as follows: $c\left(n\right)$ is defined as the complexity count of a given symbol sequence $S=(s_{1},s_{2},\cdots ,s_{n})$. $b\left(n\right)$ is the progressive behavior of a random sequence. $b\left(n\right)=\lim_{n\to \infty }c\left(n\right)=n/\log_{2}n$, and $C_{LZN}\left(n\right)=c\left(n\right)/b\left(n\right)$. As we all know, the complexity of completely random sequences tends toward one, while the regular sequence tends toward zero [27]. That is, the larger the $C_{LZ}$ is, the weaker the periodicity of the symbol sequence. The more new patterns there are, the higher the complexity becomes. As can be seen in Figure 6, the complexity of the sequence generated by the GOST algorithm we used is the highest.

#### 4.2.2. Randomness

**Figure 7:**
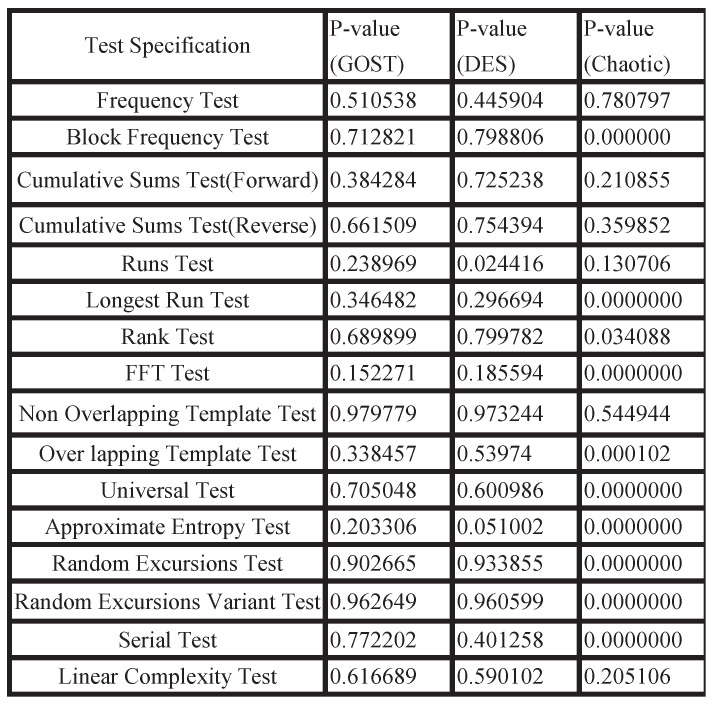
NIST test.

In practical applications, for keys, good randomness is essential for secure transmission. Therefore, we use the randomness test suite developed by the National Institute of Standards and Technology (NIST) to evaluate the randomness of the generated keys, referred to as the NIST test. The NIST test uses 16 test methods for randomness testing. The significance level α is used to characterize the randomness of the key, and it is set as 0.01. Furthermore, the *p*-value is the probability that a sequence has better randomness than a true random sequence. If the *p*-value ≥α, it means that the sequence has passed the randomness test. We use the software sts-2.1.2 to test the randomness of the generated sequence and compare it with the randomness of the sequence generated by the chaotic algorithm and the block cipher algorithm DES. It can be seen from Figure 7 that more than half of the sequences generated by the chaotic algorithm cannot exceed 0.01, so it did not pass the randomness test. Even the sequence generated by the GOST algorithm exceeds 0.05. Moreover, more than half of the *p*-value of the sequence generated by the GOST algorithm exceeds the *p*-value of the sequence generated by the DES algorithm. In summary, the key sequence generated by the proposed method has strong randomness.

## 5. Security Performance Analysis

To evaluate the security of the proposed secure OTFS scheme, we consider its resistance to some effective attacks (including brutal force, chosen-plaintext, and statistical attacks). Here, we make the following assumptions about the eavesdropper, Eve. First, for all data packets exchanged by Alice and Bob, Eve can completely overhear. Second, Eve completely understands the secret key generation algorithm. Third, Eve will not interfere with Alice and Bob.

### 5.1. Brutal Force Attacks

Brutal force attacks are also exhaustive search attacks. In this attack mode, the attacker Eve insists on trying all possible secret keys in the secret key space until the correct secret key is successfully found. In fact, when the key space is large enough, it will cause the exhaustive search to fail, thereby resisting brutal force attacks. In other words, whether an encryption system can resist brutal force attacks depends on the size of the key space. Because our key is extracted based on channel state information, the slightest change in the channel will result in a completely different key. Take the OTFS system with N=256 and M=16 as an example, the size of the IFFT matrix is 256∗256, so its key space size is 256!; the size of the FFT matrix is 16∗16, so its key space size is 16!. Therefore, the total key space of our proposed transmission mechanism is $10^{578}$. Assuming that the fastest computer, whose calculation speed is $2.5\times 10^{13}/s$ that, is used to obtain the correct secret key, the illegal eavesdropper Eve will spend $10^{556}$ years. This will cause the exhaustive search to fail, so the transmission mechanism we propose is sufficient to resist brutal force attacks.

### 5.2. Chosen-Plaintext Attacks

Assuming that the attacker, Eve, has access to the encryption machine, it can construct a ciphertext corresponding to any plaintext. Because the wireless channel has the characteristics of instantaneity and uniqueness, the encryption sequence ($D_{x}$, $D_{y}$, $D_{z}$, $D_{w}$), generated according to the wireless channel, is completely dynamic. That is, the IFFT matrix and the FFT matrix controlled by $D_{x}$, $D_{y}$, $D_{z}$, $D_{w}$ are also completely dynamic. The input OTFS symbols are encrypted into dynamic ciphertext through the IFFT matrix and the FFT matrix. The eavesdropper, Eve, must have real-time channel state information between the legitimate users, Alice and Bob, in order to process the fully dynamic ciphertext. However, Eve cannot know the channel state information between Alice and Bob, so the chosen-plaintext attack is invalid. In other words, the transmission mechanism we propose is sufficient to resist selective plaintext attacks.

### 5.3. Statistical Attacks

A statistical attack refers to the method by which the attacker, Eve, deciphers the password according to the statistical law of plaintext, ciphertext, and key. Being able to resist statistical analysis has become a basic requirement of modern cryptography. In practical applications, due to the number of encrypted OTFS symbols, the illegal eavesdropper, Eve, cannot launch statistical attacks through statistical changes in OTFS symbols. Therefore, the transmission mechanism we propose is sufficient to resist statistical attacks.

## 6. Simulation Results Discussion

In this section, we use MATLAB software for the performance simulation. To prove the overall performance of the entire system, under the mechanism of our proposed transmission scheme, we perform PAPR and Bit-Error Ratio (BER) simulations, as well as independence tests, at the same time. In this section, the simulation sets the carrier spacing to △f= 15kHz and the carrier frequency to $f_{c}=35\;\mathrm{GHz}$, and the channel fading follows the Rayleigh variables’ distribution. The number of propagation paths is two, and a 16-QAM symbol modulation is used.

### 6.1. Independence Performance

**Figure 8:**
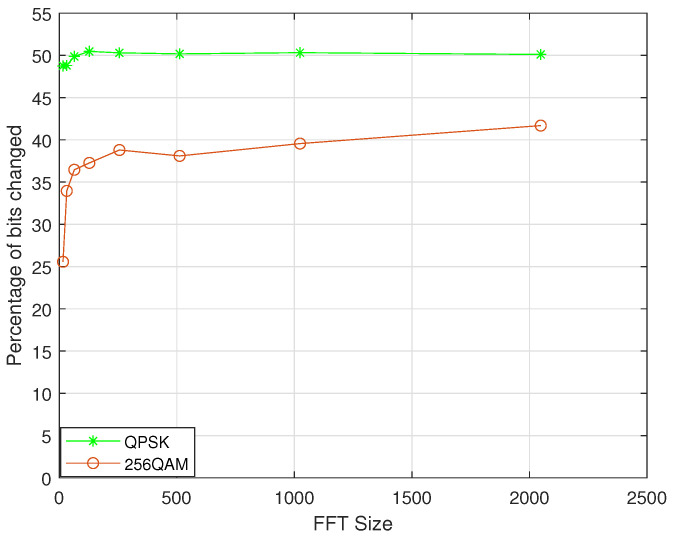
Independence test results.

The independence is the bit difference between the encrypted and original OTFS symbols. In order to ensure a secure OTFS encryption technique, it should be close to 50% [4]. It is assumed that QPSK modulation and 256QAM modulation are adopted, respectively. The vertical axis of the graph represents the percentage of the bit change between the encrypted and original OTFS symbols. It can be seen from Figure 8 that when QPSK modulation is used, the ordinate is close to 50%. When 256QAM modulation is used, the percentage of the bit change decreases slightly but remains around 40%. These results demonstrate that our scheme ensures a good cryptographic performance in terms of the difference properties. Therefore, our scheme can ensure the underlying security of the OTFS transmission.

### 6.2. PAPR Performance

**Figure 9:**
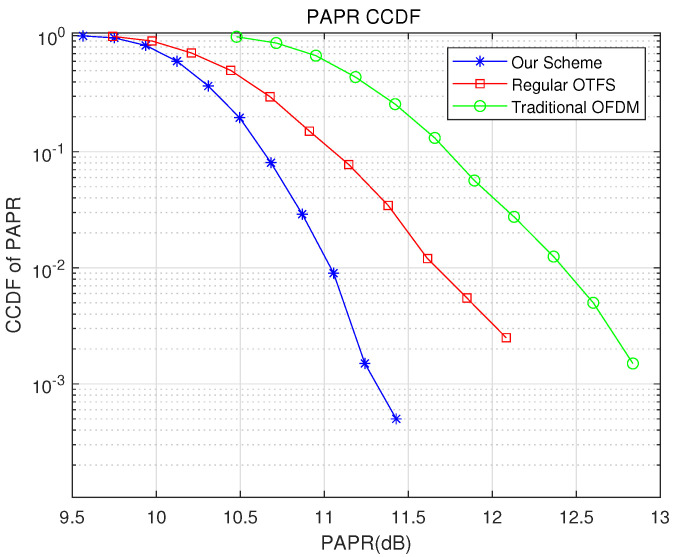
CCDF of PAPR for different schemes.

As shown in Figure 9, traditional OFDM symbols will cause a high PAPR, thereby reducing the system transmission performance. Although the conventional OTFS symbol reduces the PAPR to a certain extent, the PAPR of the transmission system is still very high. The proposed improved selective mapping algorithm can significantly reduce the PAPR of OTFS symbols with lower complexity. It can be seen from the figure that when the Complementary Cumulative Distribution Function (CCDF) is $10^{-3}$, compared with the traditional OFDM system, our proposed scheme can obtain a 2.5-dB PAPR reduction; compared with the conventional OTFS system, our proposed scheme can obtain a 1.0-dB PAPR reduction.

### 6.3. BER Performance

**Figure 10:**
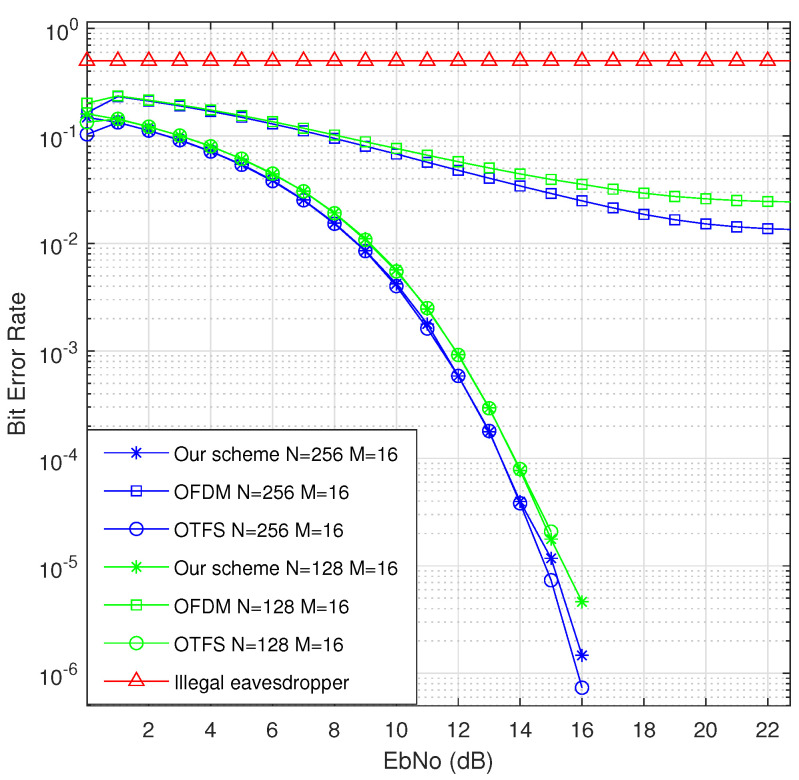
BER for different schemes.

As shown in Figure 10, compared with the conventional OTFS system, our proposed transmission mechanism will not reduce the bit-error rate of the legal receiver, Bob. Compared with the traditional OFDM system, under the same signal-to-noise ratio, the proposed transmission mechanism can significantly increase the bit-error rate difference between Bob and Eve. With the increase in the signal-to-noise ratio, the more obvious the bit-error rate difference becomes, and the more obvious the superiority of our proposed transmission mechanism becomes.

**Figure 11:**
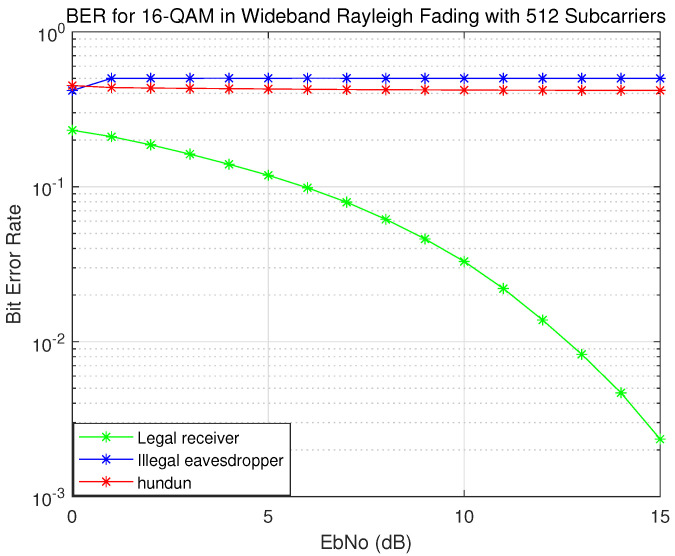
BER for legal receiver and illegal eavesdropper.

As shown in Figure 11, because the illegal eavesdropper, Eve, cannot extract the channel information between Alice and Bob, it cannot extract the correct secret key and thus cannot crack the matrix replacement process and cannot perform correct demodulation, so the bit-error rate is close to 0.5. However, due to the reciprocity of the channel, the legitimate receiver, Bob, can extract the correct secret key and demodulate correctly, so the transmission mechanism we propose will not affect Bob’s bit-error rate. For the traditional OTFS system, since the eavesdropper, Eve, knows the modulation matrix, the bit-error rate is much lower than 0.5 through correct demodulation. In the scheme using the chaotic algorithm as the key generation algorithm, there is only one source of the initial value, so the key is not sensitive enough to the channel change; though the bit-error rate is still high, it is less than 0.5. To sum up, our proposed transport mechanism has superior security performance.

## 7. Conclusions

In this paper, we have proposed a 2D permutation-aided OTFS secure transmission scheme that uses the GOST algorithm to perform disturbance control on the OTFS modulation domain. Furthermore, we have proposed an improved SLM algorithm, which can significantly improve the PAPR problem with very low complexity. We have carried out a comprehensive security analysis and simulation evaluation. Our results have shown that the transmission scheme can obtain a promising independence performance, ensuring the underlying security property of the OTFS. Furthermore, compared to the OFDM scheme, our proposed scheme can reduce the PAPR by 20% and significantly improve the BER. To facilitate practical implementation, future studies may consider the design of the OTFS secure scheme in non-reciprocal channel scenarios.

## Data Availability

Not applicable.
